# Blended-eLearning Improves Alcohol Use Care in Kenya: Pragmatic Randomized Control Trial Results and Parallel Qualitative Study Implications

**DOI:** 10.1007/s11469-022-00841-x

**Published:** 2022-08-12

**Authors:** Veronic Clair, Abednego Musau, Victoria Mutiso, Albert Tele, Katlin Atkinson, Verena Rossa-Roccor, Edna Bosire, David Ndetei, Erica Frank

**Affiliations:** 1 School of Population and Public Health, University of British Columbia, Vancouver, Canada; 2 Africa Mental Health Research and Training Foundation, Nairobi, Kenya; 3 Africa Mental Health Research and Training Foundation, Nairobi, Kenya; 4 Department of Psychiatry, University of Nairobi, Nairobi, Kenya; 5 Annenberg Physician Training Program in Addiction Medicine, Vancouver, Canada

**Keywords:** Alcohol use, screening and brief interventions, pragmatic randomized control trial, mixed-methods, qualitative research, eLearning, blended learning, human ressources for health, Low and Middle-income Countries, Kenya, stigma, Health Education, continuing medical education

## Abstract

Alcohol use is the 5th most important risk factor contributing to the global burden of diseases, with stigma and a lack of trained health workers as the main barriers to adequate care. This study assesses the impact of providing blended-eLearning courses teaching the alcohol, smoking, and substance involvement screening test (ASSIST) screening and its linked brief intervention (BI). In public and private facilities, two randomized control trials (RCTs) showed large and similar decreases in alcohol use in those receiving the BI compared to those receiving only the ASSIST feedback. Qualitative findings confirm a meaningful reduction in alcohol consumption; decrease in stigma and significant practice change, suggesting lay health workers and clinicians can learn effective interventions through blended-eLearning; and significantly improve alcohol use care in a low- and middle-income country (LMIC) context. In addition, our study provides insight into why lay health workers feedback led to a similar decrease in alcohol consumption compared to those who also received a BI by clinicians.

**Supplementary Information:**

The online version contains supplementary material available at 10.1007/s11469-022-00841-x.

The global burden of disease due to tobacco, alcohol, and other substance use is increasing and substantial. Tobacco use, alcohol use, and substance use ranked as the 2nd, 9th, and 14th highest causes of disability-adjusted life years (DALYs) worldwide, with increases in age-standardized DALY rate of 24.3% for tobacco and 37.1% for alcohol from 1990 and 2019 (Murray et al., 2020). The calculated disease burden does not include the impact of alcohol and substance use on a wide variety of social, financial, legal, and relationship problems for individuals, families, and communities (Humeniuk et al., 2010a; Ndetei et al., 2009). As such, alcohol and substance use disorders (AUD and SUD) put an enormous burden on the healthcare system and impact economic development (Patel et al., 2007). Health expenditures and access to care for mental illnesses and substance use disorders are disproportionately low compared to their associated disease burden (Vigo et al., 2016; World Health Organization, 2018). Only 10.3% of those with past-year SUDs received minimally adequate treatment in high-income countries, and only 1% in low- and middle-income countries (LMICs) (Degenhardt et al., 2017). This can change. Other sources of disease burden have decreased greatly due to concerted efforts worldwide (Murray et al., 2020). Similar concerted and strategic actions to target harm from substance use are long-overdue and have been deemed a priority by the World Health Organization (WHO) in the Mental Health Global Action Plan (mhGAP) (World Health Organization, 2010).

Integrating effective interventions such as screening and brief interventions (BIs) in primary care is needed to address the treatment gap in LMICs (Patel et al., 2015; World Health Organization, 2019). There is strong evidence supporting the use of BIs to decrease alcohol use and its associated complications and comorbidities and some evidence for other substances (Humeniuk et al., 2008, 2012; Kaner et al., 2018). However, most of these studies have been conducted in high-income countries (HICs) (Babor et al., 2013; Kaner et al., 2018; O'Donnell et al., 2013). LMICs differ greatly from HICs in terms of availability and level of training, access to primary care, general population awareness of substance use’s impacts, and in cultural norms (Awenva et al., 2010; Babor et al., 2013; Hill et al., 2021; O'Donnell et al., 2013). Some of the most rigorous studies in LMICs have been based on the alcohol, smoking, and substance involvement screening test (ASSIST)-linked BI (Humeniuk et al., 2008, 2010, 2012). However, there have been few replications studies or studies on addressing the health worker training gap identified by the WHO (World Health Organization, 2019). This highlights the need for research on effective and scalable training in LMICs and the impact and sustainability of services ensuing from those training.

Blended-eLearning (in-person plus digital) could provide effective and cost-effective training options, even in LMICs, especially as technological access improves (Bahia & Suardi, 2019; George et al., 2014; Gomez, 2014; Lewis et al., 2014; Liu et al., 2016; Maloney et al., 2012, 2015; Marrinan et al., 2015; Sandars, 2010; Sissine et al., 2014; The World Bank, 2020; Walsh et al., 2010). However, it has not been studied extensively in LMICs.

The computer-based drug and alcohol training and assessment in Kenya (eDATA-K) mixed-method research program dynamically adapted the NextGenU.org model of free eLearning to the Kenyan context and assessed training impacts (Frank et al., 2016; Galway et al., 2014; Rossa-Roccor et al., 2017). Further details on eDATA-K program of research, its methods, associated theory of change, successful trainees acquisition of substance use services related competencies, and positive impact on stigma are available in other publications (Clair et al., in press, 2016a, 2016b, 2016c, 2019, 2022; Hsiang-Te Tsuei et al., 2017).

The research questions addressed by this article are the following:

1. In patients with moderate to high alcohol-related risk levels, attending typical primary care clinics, what is the impact on alcohol consumption of receiving the ASSIST feedback by lay health workers compared to those receiving that same feedback by lay health workers in addition to a BI delivered by clinicians, with clinicians and lay health workers trained via blended-eLearning, as observed in two pragmatic randomized control trials (one in public and one in private facilities) in an LMIC context?
2. What is the experience of patients and health workers involved in those randomized control trials (RCTs)?

## Methods

Per best practices in studying complex interventions, eDATA-K used a mixed-methods developmental evaluation, including parallel qualitative data collection alongside the RCTs, to strengthen the understanding of the innovation, its implementation, and impact (Patton, 2011). The use of multiple methods triangulates the findings, therefore enhancing explanatory capacity and validity (Cathain et al., 2013; Clark et al., 2008; Kaner et al., 1999; O'Cathain et al., 2014; Spillman, 2014; Tarrow, 2004).

Ethical approvals were obtained from the University of British Columbia and the Kenya Medical Research Institute ethics boards; RCTs were registered with ClinicalTrials.gov (NCT02388243).

### Setting and Context

eDATA-K involved public and private facilities in separate RCTs, as prior studies on mental health interventions show different results between public and private settings (Patel et al., 2010). Public facilities were from two similar counties selected for their comparability and population characteristics, each including about 1 million Kenyans living in typical suburban and rural conditions. Private facilities were in Nairobi or an adjacent county. Selection criteria for the facilities were providing primary care, willingness, and ability to engage in research (electricity, staffing, sufficient patient attendance) and have a minimum of one staff who completed the screening course (lay health worker in public facilities, support staff in private facilities) and one clinician (nurse, physician or clinical officer) who completed the primary care course. The NextGenU.org courses were completed in the prior phase of eDATA-K. Eight of eleven public and two of four private facilities that participated in the training phase had enough trained health workers to participate in the RCT.

The courses included modules on the ASSIST screening tool and associated BI, communication, confidentiality, and stigma (Clair et al., 2019; Tsuei et al., 2017). ASSIST-linked BIs components considered important in generating change in substance use have been summarized with the FRAMES acronym (Humeniuk et al., 2010a):

- Feedback (personally relevant)
- Responsibility (helping people realize their need for change and that they have choices related to their substance use and behaviors)
- Advice (provision of clear advice to reduce harm from continued use, delivered in a non-judgmental manner)
- Menu of option (suggesting a range of strategies)
- Empathy
- Self-efficacy (eliciting strengths and capacity to change)

### RCT Design and Procedures

The design and procedures are based on the seminal study by Fleming et al. (1997) and illustrated in supplementary Figure S.1. Anyone 18 or older presenting to a participating facility seeking outpatient consultation, when a screener was available, was offered the general health screening consisting of the RAPA for physical activity level (Topolski et al., 2006), weight and height for nutritional status, alongside the ASSIST. The inclusion of other health issues than alcohol use masked the focus on substance use decreasing recruitment bias and stigma. Those screened received verbal feedback, with short written advice and directed to the research staff if their ASSIST score fell in the included range. Eligible participants were randomized to receive no more intervention than the simple feedback already received (Fb group) or feedback plus a BI (Fb + BI group). Study staff, independent from intervention delivery, provided the relevant instructions based on allocation from a computer-generated randomization list concealed until allocation. After randomization, only those in the Fb + BI group were instructed to show/discuss their ASSIST score with their clinician. Clinicians were instructed to provide BIs only to those presenting their scores and to offer one or two brief follow-up visits. Screeners and clinicians were blinded to the randomized allocation. Study staff were blinded at follow-up by carrying follow-up on participants from a different clinic than those they randomized. Baseline and follow-up questionnaires included demographic surveys and gold-standard, timeline follow-back methods for assessing self-reported alcohol consumption (Hjorthøj et al., 2012; Sobell & Sobell, 1992). Participants presenting for follow-up data collection were offered soap as compensation for their time.

### RCT Participants’ Recruitment

Consenting participants at moderate or high risk from alcohol use (a score ≥ 11) and ≥ 18 years old were eligible unless they met exclusion criteria. Exclusion criteria were pregnancy, having attended an alcohol treatment program in the previous year, or reporting symptoms of suicide or severe neurological/psychiatric impairment (e.g., psychosis).

### Statistical Analyses

Statistical analyses were performed through SPSS v23 and Stata v14.0, with group allocation concealed until the end result. All analyses used a 2-tailed alpha level of 0.05. Per SPSS, each RCT arm needed 146 participants to achieve 80% power using the expected change in alcohol consumption from prior studies (Kaner et al., 2007). A wealth index using principal component analysis was built from the socio-economic status (SES) survey, per best practices for LMICs (Chuma & Molyneux, 2009; Howe et al., 2012; Vyas & Kumaranayake, 2006). Independent samples *t* tests assessed unadjusted differences in alcohol consumption between groups at every time point, whereas paired *t* tests were performed to assess changes in each study group between baseline and follow-ups.

Intention-to-treat analyses were performed based on generalized estimating equation (GEE) models of the alcohol quantity consumed over the last 7 days, in grams, adjusting for SES, with appropriate imputation, taking clustering into account (Zeger et al., 1988). To investigate the effect of loss to follow-up, complete cases and imputed analysis were compared in a sensitivity analysis (Jakobsen et al., 2017).

### Qualitative Methods and Analyses

Qualitative data were analyzed through a phenomenological lens (Dowling, 2007; Norlyk & Harder, 2010; Sandelowski, 2000a, 2000b). Data was collected via field observation, focus group discussions (FGDs), and key informant interviews (KIIs). FGDs were used to encourage dialogue and joint meaning-making (Krueger, 1994; Morgan & Krueger, 1993; Powell & Single, 1996; Rabiee, 2004; Wilkinson, 1998). FGD guides included questions about their general study experience and the perceived impact of the ASSIST screenings with or without BI. (Betts et al., 1996; Krueger, 1994; Morgan & Krueger, 1993; Powell & Single, 1996; Rabiee, 2004; Sandelowski, 2000a, 2000b; Wilkinson, 1998). If selected participants could not participate in an FGD, KII was conducted.

A stratified random sampling framework was used to recruit diverse RCT participants who provided signed consent. FGDs and KIIs were audio-recorded, with co-/moderators taking written notes on body language and major immediate emerging themes and summarizing their understanding of participants’ main points. All participants received a small monetary compensation.

Kenyan moderators conducted the FGDs and KIIs in Kiswahili, other local languages, or English, according to participants’ preferences. Audio recordings were transcribed, translated, and imported into NVivo, a qualitative data management software. Latent-content analysis using a constant comparison method was used to analyze the data (Boeije, 2002).

Field observations conducted through regular site visits, and in course-related events, included recorded discussions with research staff and records of meetings and other significant interactions by qualitative research staff with participants and other staff.

Canadian and Kenyan researchers read all transcripts and independently recorded common emerging words, phrases, and themes. Similar themes were formulated into a preliminary coding framework with in-depth discussion of opposing/unique themes or interpretations. Cycles of discussion, reflection, and data immersion generated new themes until data saturation was reached and discrepancies resolved in group consultations. As insight developed, researchers jointly re-categorized data in a reductionist process to extrapolate meaning (Boeije, 2002; Dowling, 2007; Norlyk & Harder, 2010; Sandelowski, 2000a, 2000b) which was further informed by outreach to the larger research team, a subset of health workers, and key collaborators, including other research staff RAs who had numerous discussions (while collecting qualitative and quantitative data) with patients on their experience of the screening/BI.

## Results

### RCT Participants

**Fig. 1:**
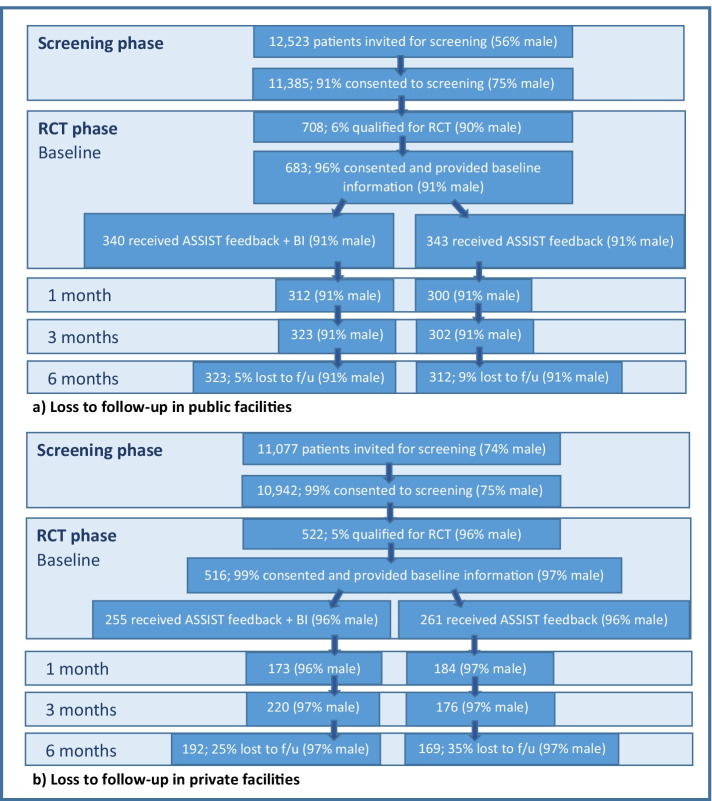
RCT participant selection and loss to follow-up

RCT patient recruitment and loss to follow-up are presented in Fig. 1. Of the 683 patients in public facilities and 516 in private facilities, respectively, 91% and 97% were male (mean age = 38.1 and 36.7 years). There were few SES differences between groups (Table 1). There were differences in wealth quintile distribution in public facilities, while age and marital status differed in private facilities. Baseline alcohol consumption was similar in Fb + BI and Fb groups in public facilities; in private facilities, it was slightly higher in the Fb + BI group.

**Table 1:** Baseline characteristics by intervention groups, public and private facilities

		**Public**			**Private**		
**Variable**		**Fb + BI**	**Fb**	***p***	**Fb + BI**	**Fb**	***p***
Gender (*n* (%))	Male	311(49.9)	312(50.1)	0.814	245(49.1)	254(50.9)	0.430
	Female	29(48.3)	31(51.7)		10(58.8)	7(41.2)	
Wealth index quintile (*n* (%))	1 poorest	86(54.1)	73(45.9)	**0.042**^**a**^	49(47.1)	55(52.9)	0.770
	2	117(49.0)	122(51.0)		54(53.5)	47(46.5)	
	3	62(55.9)	49(44.1)		51(47.7)	56(52.3)	
	4	8(26.7)	22(73.3)		65(52.0)	60(48.0)	
	5 richest	67(46.5)	77(53.5)		36(45.6)	43(54.4)	
Employment status (*n* (%))	Self-employed	97(44.5)	121(55.5)	0.166	47(46.1)	55(53.9)	0.644
	Casual labor	116(49.8)	117(50.2)		33(45.2)	40(54.8)	
	Employed	69(53.1)	61(46.9)		157(51.5)	148(48.5)	
	Unemployed	57(57.0)	43(43.0)		16(53.3)	14(46.7)	
Marital status (*n* (%))	Ever married	258(51.1)	247(48.9)	0.330	219(51.9)	203(48.1)	**0.020**^**a**^
	Single (never married)	80(46.8)	91(53.2)		35(38.5)	56(61.5)	
Education level completed (*n* (%))	Primary and below	183(50.4)	180(49.6)	0.827	54(52.4)	49(47.6)	0.799
	Secondary	111(47.8)	121(52.2)		135(48.6)	143(51.4)	
	Tertiary	27(50.0)	27(50.0)		52(49.5)	53(50.5)	
Household size (*n*; mean ± SD)		5.3 ± 3.5	5.0 ± 3.3	0.253	4.0 ± 2.4	3.7 ± 2.2	0.081
Age (years; mean ± SD)		38.5 ± 13.5	37.8 ± 12.4	0.455	37.8 ± 10.2	35.7 ± 9.9	**0.016**^**a**^
Alcohol intake t_0_ (g; mean ± SD)		422.8 ± 609.5	406.7 ± 514.0	0.709	416.2 ± 470.3	326.4 ± 413.5	**0.022**^**a**^

^a^Statistically significant at *p* < 0.05, using chi-square tests for categorical variables and independent samples *t* test for continuous or ordinal variables

### Changes in Alcohol Consumption over Time

**Fig. 2:**
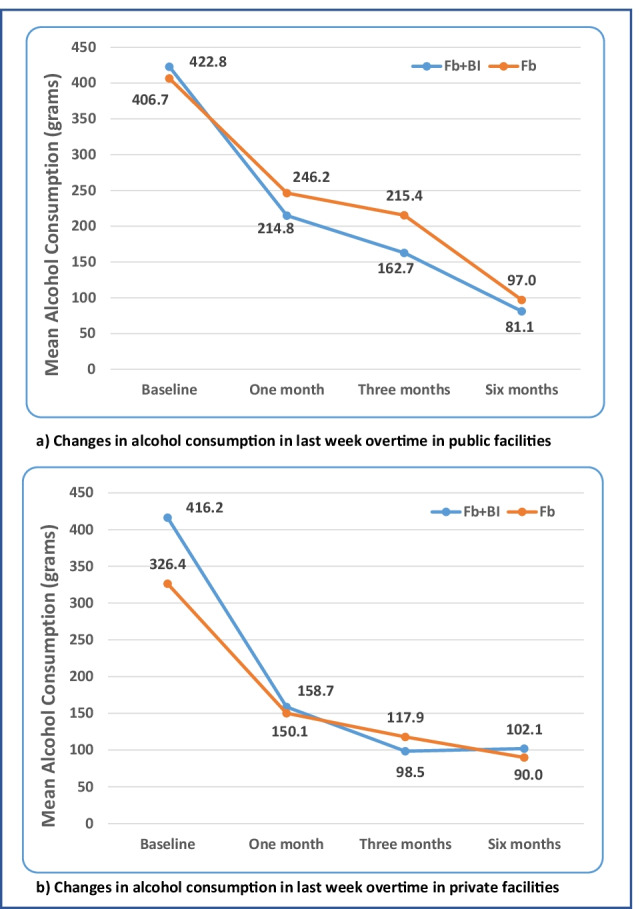
Changes in alcohol consumption in the last week over 6 months

There were statistically significant decreases in alcohol consumption, the primary outcomes of interest, at almost all time points in both public and private facilities (Fig. 2 and Table 2). The average decrease of alcohol consumed by public facility participants at 6 months was 341.6 and 309.6 g, respectively, in the Fb + BI group and Fb group. In private facilities, the reductions were 314.1 and 236.4 g. There were no statistically significant differences in the amount consumed between Fb and Fb + BI at follow-up time points (Table 3).

**Table 2:** Decrease in alcohol consumption from baseline to 6 months post-intervention in public facilities and private facilities (paired *t* test)

Treatment allocation	Time	Paired Mean Decrease (g)			*t*	*p*
		Mean	SE	95% CI		
**Public facilities**						
Fb + BI	Baseline to 1 month	208.0	36.4	(136.3; 279.7)	5.7	** < 0.001**^**a**^
	1 to 3 months	52.0	22.0	(8.7; 95.3)	2.4	**0.019**^**a**^
	3 to 6 months	81.6	15.8	(50.6; 112.7)	5.2	** < 0.001**^**a**^
Fb	Baseline to 1 month	160.5	31.8	(98.0; 222.9)	5.1	** < 0.001**^**a**^
	1 to 3 months	30.7	28.9	(− 26.1; 87.6)	1.1	0.289
	3 to 6 months	118.4	26.7	(65.8; 171.0)	4.4	** < 0.001**^**a**^
**Private facilities**						
Fb + BI	Baseline to 1 month	257.5	26.3	(205.7; 309.4)	9.8	** < 0.001**^**a**^
	1 to 3 months	60.2	11.6	(37.4; 83.0)	5.2	** < 0.001**^**a**^
	3 to 6 months	− 3.6	9.2	(− 21.7; 14.5)	− 0.4	0.696
Fb	Baseline to 1 month	176.3	22.1	(132.7; 219.9)	8.0	** < 0.001**^**a**^
	1 to 3 months	32.2	11.8	(9.0; 55.4)	2.7	**0.007**^**a**^
	3 to 6 months	27.9	9.0	(10.2; 45.7)	3.1	**0.002**^**a**^

^a^Statistically significant at *p* < 0.05

**Table 3:** Difference in alcohol consumption between treatment groups for each time point (*t* test)

Time	Treatment allocation	Mean (g)	SD	t	df	*p*
**Public facilities**						
Baseline	Fb + BI	422.8	609.5	0.37	681	0.709
	Fb	406.7	514.0			
1 month	Fb + BI	214.8	323.4	-1.29	681	0.196
	Fb	246.2	311.4			
3 months	Fb + BI	162.7	296.5	-1.64	681	0.102
	Fb	215.4	515.7			
6 months	Fb + BI	81.1	137.6	-1.32	681	0.189
	Fb	97.0	176.2			
**Private facilities**						
Baseline	Fb + BI	416.2	470.3	2.306	514	**0.022**^**a**^
	Fb	326.4	413.5			
1 month	Fb + BI	158.7	204.2	0.490	514	0.624
	Fb	150.1	194.0			
3 months	Fb + BI	98.5	130.0	-1.702	514	0.089
	Fb	117.9	129.2			
6 months	Fb + BI	102.1	145.1	1.076	514	0.283
	Fb	90.0	108.2			

^a^Statistically significant at p < 0.05

Similarly, the imputed GEE results, adjusted for SES (Table 4), still showed that alcohol consumption reduced significantly over time in public and private facilities, with similar decreases in the Fb + BI and Fb groups in both facility types. These results persisted in sensitivity analysis with raw data (Supplementary Table S.1).

**Table 4:** Socioeconomic status and changes in alcohol consumption in public and private facilities (GEE)

Variable	Category	Public					Private				
		**β**	**S.E**	**95% confidence interval**		***p***	***β***	**S.E**	**95% confidence interval**		***p***
				**Lower**	**Upper**				**Lower**	**Upper**	
Treatment allocation	Fb + BI	9.54	47.51	− 83.58	102.66	0.841^**a**^	74.81	42.5	− 8.47	158.09	0.078
	Fb	Ref					Ref				
Gender	Male	48.75	20.20	9.17	88.34	**0.016**^**a**^	104.31	32.1	41.43	167.20	**0.001**^**a**^
	Female	Ref					Ref				
Wealth index quintiles	1 poorest	− 66.22	22.18	− 109.69	− 22.74	**0.003**^**a**^	46.49	23.4	0.64	92.34	**0.047**^**a**^
	2	− 28.69	23.19	− 74.15	16.77	0.216	53.21	20.1	13.82	92.60	**0.008**^**a**^
	3	22.89	28.03	− 32.04	77.83	0.414	90.02	23.5	43.92	136.13	**0.000**^**a**^
	4	78.62	84.54	− 87.08	244.33	0.352	26.55	17.5	− 7.68	60.77	0.128
	5 richest	Ref					Ref				
Employment status	Self-employed	20.71	25.15	− 28.58	70.00	0.410	− 54.83	53.2	− 159.06	49.40	0.303
	Casual labor	51.47	24.90	2.67	100.26	**0.039**^**a**^	− 28.00	58.7	− 143.00	87.00	0.633
	Employed	29.88	26.52	− 22.09	81.85	0.260	− 96.65	51.8	− 198.20	4.90	0.062
	Unemployed	Ref					Ref				
Marital status	Ever married	16.61	22.46	− 27.40	60.63	0.459	72.37	23.5	26.38	118.35	**0.002**^**a**^
	Single	Ref					Ref				
Education level completed	Primary or less	1.47	33.28	− 63.75	66.69	0.965	35.97	27.4	− 17.76	89.71	0.189
	Secondary	− 19.54	32.91	− 84.05	44.96	0.553	− 8.03	18.3	− 43.95	27.90	0.661
	Tertiary	Ref					Ref				
Household size		− 5.73	2.08	− 9.81	− 1.65	**0.006**^**a**^	− 0.77	3.5	− 7.56	6.01	0.823
Age (years)		− 0.29	0.74	− 1.75	1.16	0.691	− 3.30	1.0	− 5.18	− 1.41	**0.001**^**a**^
Time		− 97.83	9.73	− 116.90	− 78.75	** < 0.001**^**a**^	− 72.04	8.4	− 88.47	− 55.61	** < 0.001**^**a**^
Fb + BI*Time		− 11.00	14.88	− 40.16	18.16	0.460	− 21.31	12.2	− 45.29	2.67	0.082
Fb*Time		Ref					Ref				

^a^Statistically significant at *p* < 0.05, *GEE* generalized estimating equations with imputed data, using ten randomly generated imputed values for missing alcohol quantity consumed at follow-up, taking into account SES characteristics, allocated group, and baseline quantity of alcohol consumed. Given the clustered nature of the data (participants clustered within clinics and clinicians), we computed robust variance estimators for all GEE analyses

Statistically significant covariates included gender, wealth, household size, and age (Table 4). Male participants decreased less in both types of facilities. In public facilities, participants on the poorest quintile decreased more than the wealthiest, while in private facilities, participants of the lowest three quintiles reduced less than the wealthiest quintile. The larger the household size, the larger the decrease in alcohol consumption in public facilities. An increase in age was associated with a decrease in private facilities.

### Participating Facilities and Health Workers

The FGDs and KIIs with patients included 86 participants, and those with health worker had 31, for a total of 117 (Table 5). Participating public facilities included two outpatient departments and seven community clinics involving 24 clinicians (70% female) and 24 screeners (58% female), with 13 (54%) of each group took part in FGDs or KIIs. Participating private facilities included one private clinic and one private hospital outpatient department involving 5 clinicians (25% female) and 9 screeners (56% female), of those 3 clinicians (60%) and 2 screeners (22%) participated in the FGDs or KIIs.

**Table 5:** FGD and KII participants

	**Number of participants**		
**RCT participants**	**Male**	**Female**	**Total**
***Public facilities, county 1***	*19*	*20*	*39*
FGD men, Fb + BI	9		
FGD men, Fb	10		
FGD women, Fb + BI		10	
FGD women, Fb		11	
***Public facilities, county 2***	*23*	*4*	*27*
FGD men, Fb + BI	11		
FGD men, Fb	12		
KIIs woman, Fb + BI		2	
KIIs woman, Fb		2	
***Private facilities***	*19*		
FGD men, Fb + BI	11		
FGD men, Fb	8		
**Total**	**49**	**37**	**86**
**Healthcare workers**			
***County 1 (5 facilities)***			
FGD clinicians	3	5	
FGD screeners	2	6	
KII clinician	1	1	
***County 2 (3 facilities)***			
FGD clinicians (3) and screeners (5)	*2*	*6*	
***Private facilities (2 facilities)***			
FGD clinicians (2) and screeners (2)	3	1	
KII clinician		1	
**Total**	**11**	**20**	**31**
**Grand total**	**60**	**57**	**117**

### Qualitative Findings

The iterative process of thematic and latent content analysis, with constant comparison, of the data from the FGDs and KIIs plus field observations, confirmed the unexpected finding of a similar and meaningful decrease in alcohol use in both groups and revealed several likely thematic etiologic mechanisms (Tables 6–9): (1) meaningful impact on alcohol consumption, (2) fundamental shift in knowledge, attitude, and practice by health worker, (3) contributing factors to screeners delivering the FRAMES components of BIs, and (4) other research design elements supporting change.

**Table 6:** Theme 1: Report of meaningful decrease in alcohol consumption in: examples from both RCT groups

RCT participants
“…when I met these people, they explained to me, they gave me papers to go and study from home. They also asked me to come after a month. I was asked if I read the papers, and I was asked whether I had reformed, and I told them I will reform with time… With alcohol, I stopped at once… They told me the dangers of alcohol, and I saw it… So, these counselling guys are like pastors who preach the word of God… Even my body, I feel like I am now healthy. Thank you very much.” FGD men, Fb, county 2 “It has changed and now [I] have stopped to drink completely.” FGD women, Fb, county 1 “I have learnt so much because now I can stay the whole day without going to drink. That is why I feel that [I] have improved very much. I also reach home early and find when food is still hot.” FGD men, Fb + BI, county 1 “So, the second time when they called me, I decided to reduce taking alcohol and as time went by, I stopped completely. … I just want this study to reach the whole of Kenya because I have seen its impacts on me.” FGD men, Fb + BI, county 2 “I have completely reduced for almost three months now. I have not even tasted alcohol.” KII women 2 Fb + BI, county 2 “I reduced my consumption of alcohol; I have actually almost dropped it.” FGD men Fb + BI private
Healthcare workers
“… you pass by and they are like, ‘he is the one who told us to stop taking alcohol.’ Can you see how we have changed?” FGD screeners, county 1 “They were coming for something else different, but after a few periods and also giving them the materials to go and read, it really helped … Actually like 90% of the patients returned and they used to say like, ‘I have quit.’ Even like today, you meet some of them in the street, they say doctor you helped me.” FGD clinicians, county 1 “At the health center, they appreciate your help for changing our community. The rate of taking alcohol has greatly changed… When we started the program, we had to teach them…Since then, those who appreciate the help have not stopped coming back for advice and help. 50%-60% were positive about the discussion and we are glad.” FGD clinicians and screeners, county 2 “I can say that this project has helped many in that some have reduced the consumption of alcohol while others have completely stopped.” FGD clinicians and screeners, county 2 “… they would say thank you for introducing us to the study, so the attitude changed. They have also reduced taking substances. Some of the patients we met at first, they look different; they are different people after the exercise. I believe it is an exercise that has brought changes even at their families. There are some who now bring their friends for the same thing.” FGD clinicians and screeners, private “So, with the brief intervention, patients were able to stop or reduce substance abuse and we could see an improvement in their health. That is an important issue because people who are living with HIV and using drugs are at a higher risk of dying as compared to those with HIV who are not using alcohol and other substances.” FGD clinicians and screeners, private “And they say [the patients] they really appreciate it, and it has been like two or three months and they have really cut down smoking, drinking. So, for some it really worked.” KII clinician 1, private

**Table 7:** Theme 2: Fundamental shift in knowledge, attitude, and practice by HCW

**Subtheme 2.A. New confidential, empathic services with decreased stigma, attracting patients**
**RCT participants**
“I thank this clinic because it has helped me. When I was called for the first time, I refused, but when I entered to see the doctor, I did not expect the doctor to talk to me the way he did. He asked me questions so well about substances which I answered… So, the doctor talked to me so nicely…so I continued with the doctor’s advice until I stopped completely taking the substances.” FGD men, Fb, county 2 “if you saw the doctor, he was…someone whom you could confide with while talking to him. The whole team was also willing to help us and this encouraged us to feel comfortable.” FGD men, Fb, private “Even the doctors were ignoring me, but the way your team handled me, I decided to change.” FGD Men, Fb + BI, private "And the way they approached us, it didn’t seem like they could disclose that secret outside. So, I accepted, and they proved my gut feelings right.” FGD women, Fb, county 1 “Yes, because I was asked questions in a private room. Even if someone else came into the room, they could wait and pause first until the person left, then we could continue. She even told me that I should be transparent so that we can help each other. I see them as helpful people and they did it very well and my privacy was kept.” FGD men, Fb + BI, county 2 “I believed them, because one, they called me alone, second, they were people who were talking politely and third I found them to be people who would keep secrets.” FGD women, Fb + BI, county 2
**Healthcare workers**
“I would say there is great attitude change because, am not bragging because I am from M…but you could see patients coming all the way from W and X…when you ask why they have travelled so far to come here…they say that they are well received here, we handle them well and that we understand them. We don’t judge them… We have a big turn up at the hospital, and it is because of mind and attitude change towards the patient.” FGD clinicians, county 1 “Yeah, the stigma is decreasing because, if I take an example of a facility here before this program came into being, whenever we got maybe a patient who is drunk, then you would find that the treatment was not so nice. Maybe most of the people would think he is an intruder within the institution; he is a troublemaker. … so, they are not given equal treatments compared to the other clients who are coming to seek treatment.” KII clinician 1, county 1 “At first, I can say there was stigma, even you could see a client, him just rush away. We don’t want to see mental people but, in the process, they came, they realized we are there to help them. So, we became friends, and the screening was so much easier than at the beginning” FGD screeners, county 1 “To us, it is an added advantage to associate with the less fortunate in the society. The name of the hospital becomes raised and kind of famous. More people are now coming to the hospital, and when they leave, they are happy.” FGD clinicians and screeners, county 2 “Men were always open, and after visiting, they could come back with their friends.” FGD clinicians and screeners, county 2 “We also changed our personal attitudes towards SUD patients, unlike the way we looked at them as failures before we were trained.” FGD of clinicians and screeners, private; with the group signifying general agreement afterward “for some, they actually bring their relatives, because you see you actually had that impact in the society, so you see some of the clients actually came back with their relatives” KII clinician, private
**Subtheme 2.B. Provision of other ASSIST-linked BI FRAMES components** ***2.B.i. Feedback, responsibility and advice elements***
**Healthcare workers**
“Like this part where you come to discover so many people are in the drinking team because they have never been told, they don’t have the knowledge at hand. They are not aware…you know it’s like there are no counselors… You find that at least with this knowledge, if everybody else is aware about this, they will involve themselves in other activities like football or some other income-generating projects.” FGD screener, county 1 “They realized that some S.U.D has led them to such grievous deeds and they started thinking about their lives.” FGD clinicians, county 1 “From my understanding, the danger of taking alcohol has been discussed openly and we have shared with the clients positively.” FGD clinicians and screeners, county 2 “…we educated them on the benefits and why they need to go through the system and once they were discovered to have this disorder, we would see what we can do to help them to complete the process.” A screener from the FGD clinicians and screeners, private
**RCT participants**
“When I was told about my score, I felt good because I accepted that state, and got to realize that I needed help. It was not something that I could be happy about, but those results were good because they made me to accept and decide that I needed to change.” FGD men, Fb, county 1 “I understood that the more you continue to drink excessively is the more you continue to spoil things like your liver” FGD women, Fb, county 1 “They told me the dangers of alcohol and I saw it. I even used to insult my wife while drunk, but right now, it is like I got saved. I am peaceful at home.” FGD men, Fb, county 2 “The information was adequate because it helped me a lot… I have learnt that alcohol is not good because it will influence someone to do bad things which might lead to family breakups.” KII Women 1 Fb County 2 “First of all, we were taught on that substance use is not a good thing and the moment you continue using them, they will affect your health.” FGD men, Fb, private “I also felt bad because nobody has been buying these substances for me, I have been buying them for myself and I felt like my money has wasted me. So, that score really hurt me and what I have been doing to myself.” FGD men, Fb, private “What I learnt is that when I am under the influence of drugs, I will not make a good decision. But when I am sober, even if I don’t make a decision, I will still be ok.” FGD men, Fb, private “So, the counseling helped me a lot… This worked out so well. He told me the consequences of alcohol consumption and how it affected me and my family.” FGD men Fb + BI, county 2 “My score is what made me to change…that is when I saw the need to change. For one, I found that I was almost beyond life. I decided to change… Even the doctors were ignoring me, but the way your team handled me, I decided to change.” FGD men, Fb + BI, private “Yes, that was the start of knowing myself. It is like I did not know about myself, but after sitting down with these people and after they gave me my score, that is the time I realized how far the substances I have been using had ruined me. So that was my turning point… the more teachings I got, the more I decided to change.” FGD men, Fb + BI, private
***2.B.ii. Use of the menu of option FRAMES component to decrease harm from alcohol use***
**RCT participants**
“It made me to limit my things even when I go out. I know that when it reaches eleven, I know that am supposed to go home.” FGD women, Fb, county 1 — changing the timing of the drinking, reducing harm “You know us, we call it “Kitindo” meaning when you stay with drunkards all the time, you will be influenced to go back to drinking. So, I have decided not to keep such companies now.” KII women 1, Fb, county 2 — changing social network “You see, if you are idle, you will go to drinking, but if you are busy, you will not.” FGD men, Fb, county 2 — occupy oneself “again, like my parents were so impressed with this study and when they started seeing the changes in me, they were more than willing to give me transport whenever I wanted to go for follow-ups, they were even wondering if they could rent an apartment for me near the hospital… (all laughing).” FGD men, Fb, private — support from loved ones “I am also too busy with my life which was so messy. I do my general cleaning over the weekend; I can visit my family and parents and I can get to work at the right time.” FGD men, Fb, private — finding activities to occupy oneself “Avoiding bad companies.” FGD men, Fb, private — changing social network “Those things of going to the bar and buy beer for everybody, even spending the money that was supposed to be for the children, have stopped. Like he has said, the best thing is we avoid going to those places.” FGD men, Fb + BI, county 1 — avoiding triggering locations, different use of money “I go home straight, and if am unable to go, I call him and he comes to fetch me.” FGD women, Fb + BI, county 1 — avoiding triggering locations, getting support from loved ones “It means being busy in groups, meetings and work. Not coming from work and then you just sit until you start thinking ‘let me go and do one two with friends. When you come from work, you have your own programs, to maybe do this and that or attend meetings, then come back… keep myself busy because if you stay idle, you can easily fall back there.” FGD women, Fb + BI, county 1 — finding activities to occupy oneself “I have completely stopped. I even meet with my friends who we used to drink together, and they ask me, “so can I buy you one today,” and I say I am saved… The third thing is I dropped all my friends who we used to drink with.” KII women 1, Fb + BI, county 2 — refusing offers to drink, changing social network “Keeping myself busy always, if I am not at the market am at home washing clothes or at the farm digging, as in making sure I am occupied every minute of the day.” KII women 2, Fb + BI, county 2 — finding activities to occupy oneself instead of drinking “So, to me shortly I can say that what I learnt is about…the first day I met with your doctor, he told me a simple term that "bad company collapses good morals". He told me so. He told me that I am very young, and he asked me to put what he had told me in mind. That encouraged me to learn more. Next, I learnt to be soft and giving people who surround me respect.” FGD men, Fb + BI, private — changing social network “Avoiding bad companies.” FGD men, Fb + BI, private — changing social network “Just to keep myself busy, like by watching movies.” FGD men, Fb + BI, private — finding activities to occupy oneself other than drinking
***Subtheme 2.B.iii. Self-efficacy***
**RCT participants**
“I used to be so drunk and even used to sleep outside in the marketplace. They also gave me soaps and it helped me because I never used to do washing or even wash my children’s clothes, I used to be so dirty. I also never used to cook for my children. I was so bad. Even my body was unhealthy; I can’t even tell you how I looked like. But right now, I can do everything.” KII women 1, Fb, county 2 “Initially, I could not even educate my children well, but right now, I can see my expectation is getting met because of the counseling I have received. I was advised to stop taking alcohol and I did. I have my projects such as chicken, cows, something that I never used to do before. So, I thank this study.” FGD men, Fb, county 2 “It has helped my family relationship with my wife and children. Also, my body is healthy now. I can say that I thank you so much because now I have a vision with my life.” FGD men, Fb, county 2 “My expectations were that if I go there, at least I can be accountable and responsible in using my money and this is something that I can see has changed and helped me in my life.” FGD men, Fb, private “I was very happy to be interviewed and asked questions about alcohol, cigarettes, and after that, I managed to stay for one week without drinking and also reduced smoking by smoking two cigarettes. That gave me the moral to continue, and I saw that if I continue like that, I can someday be able to stop all these things” FGD men, Fb + BI, county 1 “You also start liking yourself you know if you are an addict it reaches a point that you lose your self-esteem, you hate yourself so, if it is positive after the change you start loving yourself and start seeing yourself like somebody” FGD women, Fb + BI, county 1 “But after these services came and I got the knowledge, I started reducing, especially drinking in the morning. It was difficult, but I just told myself to try my best and do it.” FGD men, Fb + BI, county 1 “Yes, the more teachings I got, the more I decided to change. There is a time I came and my results had improved and this made me feel that I was doing well. So, to me, the attitude went on changing with time.” FGD men, Fb + BI, private “I came to realize that alcohol is not porridge; you don’t just drink just like porridge that is getting finished. Breweries are busy making more alcohol. So, I learnt how to control myself, and even if I consume alcohol, I now know the amount that will not affect my brain or my lifestyle… So, I have learnt that if someone decided not to take something, they can achieve it.” FGD men, Fb, private “I learnt that if someone cannot do anything, it doesn’t mean that myself I cannot as well do anything. Or if someone else cannot stop drinking, that does not mean that I cannot stop drinking.” FGD Men Fb + BI Private “Yes, and if someone decides to stop them, it is not too late. Even if someone is an addict, it is not too late.” FGD men, Fb, private

**Table 8:** Theme 3: Contributing factors to screeners delivering the FRAMES components of BIs

*Subtheme C: HCWs personal motivation to learn and help those affected by alcohol use*
“I personally I have a personal motivation. I have a brother who is a drinker or he takes alcohol and I really felt for him and I wanted to assist him with the whole family, so I wanted to learn more about how to go about his thing so that I can apply the knowledge” FGD screeners, county 1
“Yeah to me, I wanted to acquire knowledge to help my community because surely in my community, we have many people who are drunk. So, I wanted to acquire that knowledge so that I can go talk about it.” FGD screeners, county 1
“Mine like now I have a friend who uses drugs and my interest is like when we are learning about substance abuse, I was interested to apply the knowledge I have acquired in practice support and substance abuse and then I put it into practice to help the family members and the entire community to change their lifestyle.” FGD screeners, county 1
“I am very happy about it since I have the knowledge and I can be able to share with the community. Through sharing, which could happen when I attend church meetings, I am able to assist both the young and older people. The other day I was at K. and was talking to around 300 youths and they had a lot of questions, and I am very pleased about the knowledge I have acquired through training and I feel I have a lot of confidence.” KII clinician 2, county 1
“Let’s say when we are here in the hospital, they believe in us according to the relationship we have outside the hospital. When in the hospital, we interact with many people and happen to socialize with them. That is an added advantage especially to the screeners because we don ‘t come from far, so those who meet with during the screening are our dear people.” FGD clinicians and screeners, county 2
“We appreciate the knowledge because we can now handle the drunkards in a well-known manner.” FGD clinicians and screeners, county 2
“Overall, we gained education or knowledge gain, experience with dealing with SUD patients and we were able to intervene on these clients and I think the core objective we achieved.” FGD clinicians and screeners, private
“Ok, let me start with the community, the patients and the clients for them—they always see you as someone who is helping them, then the most encouraging thing to them is that they see like you are putting more effort in helping. Some actually say you helped my family because now I would be drowning somewhere else, some come with their spouse and they say you really had an impact on him and they are like we have tried this and this at home, but it hadn’t worked but with the knowledge from the hospital and that kind of a thing. Some are really grateful, and they see you as a counsellor or that kind of a thing, so others are really open and they come and share their stories with you.” KII clinician, private
“Some of these people are our family, friends and whenever we meet, someone has a smile on the face, they can shake your hand and tell you oh… I reduced taking the substances actually, that thing was helping and so on. So, there was a general health improvement.” FGD clinicians and screeners, private
“Yes, a lot, most people see us as the most educated, even with our colleagues here, they see us as people with extra knowledge.” FGD clinicians and screeners, private

**Table 9:** Theme 4: Other research design element supporting change

*Subtheme 4.A. Repeated assessments by research staff, with some supporting RCT participants*
**RCT participants**
“…and then she forwarded me to the research assistants who talked to me and advised me on drugs.” KII women 2, Fb, county 2 “even when they meet me on the road, they advise me, ask me to read the papers. Up to date, they advise me and even gave me soaps. I thank you so much” FGD men, Fb, county 2 “They were organized in that after the screening, they took us to the research assistant who even showed us the doctors who may help us, and if you saw the doctor, he was a clean person and someone whom you could confide with while talking to him. The whole team were also willing to help us and this encouraged us to feel comfortable.” FGD men, Fb, private “The whole team were also willing to help us and this encouraged us to feel comfortable.” FGD men, Fb, private “No, there were times I would even come without being told to come and I wouldn’t even call them [the research staffs] and I would get them in the office. Then I would knock on the door and get in and say hi and time them I have come for lunch; we joke for a while and then I tell them that there is a question I wanted to ask and that’s why I have come and they would welcome me. So I would ask and they would explain to me well and I would feel satisfied, then they would ask me if I have been satisfied or not and I would tell them I am satisfied and if I am not I should hide it, I should tell them so that they can look for a solution.” KII women, Fb + BI, county 2 Facilitator (F): “So they were giving you advice well, and that’s why you kept coming.” Respondent (R): “Yeah, even when we meet at the roadside, we would talk well.” KII women 2, Fb + BI, county 2 “…there were times I would even come without being told to come, and I wouldn’t even call them [the research staffs], and I would get them in the office. Then I would knock on the door and get in and say hi… we joke for a while, and then I tell them that there is a question I wanted to ask and that’s why I have come, and they would welcome me. So I would ask, and they would explain to me well, and I would feel satisfied, if I am not I shouldn’t hide it, I should tell them so that they can look for a solution.” KII women 2, Fb + BI, county 2
**Research staffs** (field observation, with audio recording)
“Facilitator (F): And when you were trying to encourage them to drink less, what were you doing to encourage them to drink less?…Or were you just asking them the question about how much they were drinking…? Respondent (R): Encouraging them to drink less to continuously drink less until they stop it.” RS county 1 “The community really accepted us, and people would bring their husbands they would cooperate and we would help them where we could.” RS county 1 “Okay, personally, I, yeah, it was satisfying. It was very satisfying to me when someone comes for follow up and they are very excited to see you to give you the good news how they've improved. Yeah, making friends. We also made friends from the people we recruited. Yeah. So yeah. Socially, my social skills also improved. Yeah.” RS county 2 “F: And did you feel participating in the project, changed your life in any way. R: Participating in the project changed my way in that I can talk to someone who is a drunkard and change him a bit. Yeah.” FGD RS county 2 “F:… and did it help you in any way for yourself to participate in this project? R: Yes, socializing and even talking to people to change their behaviors.” RS county 2

#### Theme 1. Meaningful Impact on Alcohol Use and Other Domains Reported by All Groups

RCT patient participants in both groups reported reduced alcohol consumption with many improvements in mental and physical health and in other dimensions of their lives, which they attributed to their participation in the study, per Table 6 and these quotes:

Like before, I would say that I cannot watch a game without drinking, but right now, I can watch without any alcohol. Initially, it is like I cannot see drinks in a bar and just sit but nowadays, I can just sit next to them without touching. I am also too busy with my life which was so messy, I do my general cleaning over the weekend, I can visit my family and parents and I can get to work at the right time. (FGD men, Fb, private facilities)

Since I was married, I had never had a child with my husband, that is why am saying it is very bad [alcohol]. We have stayed for long without me getting pregnant. I have been drinking a lot. After I joined this study, I stopped completely taking alcohol and I became pregnant immediately. This child I am holding here is as a result of this study… (FGD women, Fb+BI, county 1

Corroborating these patients’ reports, screeners and clinicians noticed similar impacts (Table 6).

#### Theme 2: Fundamental Shift in Knowledge, Attitude, and Practice by Health Worker

This theme, divided into several subthemes, provides evidence of the changes and contributing factors to those changes, as presented below and in Table 7.

##### Subtheme 2.A. New Confidential, Empathic Services with Decreased Stigma, Attracting Patients

 Prior to eDATA-K, neither public nor private facilities’ roles included addressing risky levels of alcohol use, according to field observations and discussions with the county and facility leadership, as well as health workers themselves at the beginning of the project. Private insurance policies specifically excluded medical care coverage for those who abused alcohol. Health workers fundamentally shifted their approach and attitudes toward people presenting with risky levels of alcohol use, from denying or delaying services to welcoming them and providing empathic assessment and interventions for the first time in these communities. Our results support that health workers developed self-efficacy and offered new evidence-based, non-judgmental, empathic, and confidential alcohol and substance use services, as well as providing better general care to those patients.

…you know that is something that is disturbing you, but you can't stop. So, if you find somebody that is willing to help you and is telling you that he is helping you and that the information is confidential, OK, you will feel uneasy at first, but if you see that it is helping you, then you become relaxed. (FGD men, Fb+BI, county 1)

I have gained knowledge and that knowledge is power… For example, my father used to take a lot of alcohol, and at one point, he happened to divorce my mother. So, I used to despise the people who take alcohol because a lot happened to me. When they come, I never attend to them... They could wait all day until another person comes in the afternoon shift to attend to them… After being mentored at Africa Mental, I discovered that these are people we can still take care of. At first, maybe it's because I didn't know how to approach them, I didn't know what to tell them, etc. Right now, I've learnt a lot and I know how to handle them better. (FGD clinicians and screeners, county 2)

Accordingly, patients in both groups reported exposure, for the first time, to new knowledge, empathy, and confidential feedback from health workers, which they appreciated and found transformative. As such, the facilities started attracting patients seeking these new services in particular:

I can say that there is a lot of change in the sense that like for instance, you find that me working in the facility, you find patients or clients coming in even when them they are not [from] around… I have seen it, so you will find that maybe some have been screened, others have heard it from their fellows who had been screened, you know, in the community. So, you find them bringing themselves, you know, to the facility. They want to know more. (FGD screeners, county 1)

##### Subtheme 2.B Provision of Other ASSIST-Linked BI FRAMES Components

 Our finding supports the widespread delivery of all the FRAMES components of effective BI by clinicians and screeners. Provision of E, empathy, is documented in subtheme 2.A. Provision of the feedback, responsibility, and advice components is illustrated in these quotes:

If you realize someone is taking that, it is good to advise them on the consequences. Then you let them make the decision. (FGD screeners, county 1)

So, when I came here, I was just so happy, we talked and if there was anything to be shared, we did and they gave me advice. They told me the disadvantages of drugs and I saw there was not any advantage of taking alcohol. So, I can say they were ok. We talked as sisters and brothers. (KII woman, Fb, county 1 #2)

Participants in all groups gave examples of various strategies used to decrease their alcohol consumption, corresponding to the “Menu of option” (M). Strategies most frequently mentioned were finding activities to occupy oneself instead of drinking and changing social networks. Other strategies included avoiding triggering locations, using money differently, getting support from loved ones or spirituality, committing to change, refusing offers to drink, reducing slowly to quit, changing the timing of the start or end of the drinking, reviewing the information provided, and reminding oneself of the negative consequence of drinking. Also, in all groups, RCT participants expressed developing self-efficacy:

Like when I came for counselling, I think it was the second time the nurse that was attending to me, we were talking about things like self-esteem, how to stop hating yourself. She even helped me on how to be positive in life, so I started seeing myself as worthy and that I can also be somebody in life. (FGD women, Fb+BI, county 1)

Participants in the Fb + BI group elaborated more on their strategies than the Fb participants. Some clinicians, but not screeners, reported consciously building self-efficacy in their patients.

#### Theme 3: Contributing Factors to Screeners Delivering the FRAMES Components of BIs

##### Subtheme 3.A. The ASSIST Tool Design

 Field observation revealed that a major contributing factor to the effective delivery of the Feedback element is that it was integrated into the ASSIST questionnaire and feedback form by design. The ASSIST-linked BI manual states p.11:

…feedback is the provision of personally relevant information which is pertinent to the client, and is delivered by the health worker in an objective way. ‘Much of the feedback given in an ASSIST-linked BI can be delivered by reading directly from the ASSIST feedback report card.’ (Humeniuk et al., 2010a)

Questions embedded in the ASSIST and information in the report card are designed to increase insight into one’s level of use and associated consequences, classifying respondents’ score in terms of low, moderate, or high-risk levels for each substance category with specific examples of health consequences. As the ASSIST questionnaire was administered by the screeners, including the provision of the feedback with the report card, it contributed to Fb group participants receiving this key element of BIs.

The Responsibility component, described as “Communicating with clients in terms such as … ‘How concerned are you by your score?’ enables the client to retain personal control over their behaviour and its consequences and the direction of the intervention” p.12 (Humeniuk et al., 2010b). As that question is asked in the feedback form, again by design, the screeners included this other FRAMES component in their patients’ interactions.

##### Subtheme 3.B Opportunities for Screeners to Learn and Provide all the FRAMES Components

 Field observations revealed that screeners had the opportunity to develop BI skills above and beyond administering the ASSIST screening. As part of the course, clinicians and screeners observed a demonstration of effective and ineffective screening and BI. Furthermore, many screening course trainees were exposed to the BI modules of the primary care course:

Also, the screening course trainees and the primary care management trainees frequently completed the training together, advancing through the module as a group in the different facilities. This exposed the trainees of the screening course to some of the primary care management modules. (FGD clinicians, county 1)

This was due to access to one computer in most facilities, with screeners and clinicians being available at similar times to complete the training (before/after work or during breaks). Additionally, in each clinic, a copy of recommended BI steps was posted to support clinicians, and a printed copy of the self-help manual was available. With screeners motivated to help the patients, they reported accessing those.

##### Subtheme 3.C. Health Workers Personal Motivation to Learn and Help Those Affected by Alcohol Use

 Trainees who completed either course were very motivated to do so because they desire to help others in their family and community. As such, they wanted to provide as much support as they could to those with whom they interacted. Screeners did not see their role in eDATA-K as research staff administering a tool to assess study eligibility but as concerned health workers wanting to help address an issue in their community. They derived personal satisfaction from the improvement they saw — encouraging them to continue without being asked to do so (Table 8):

Maybe I like learning new things, adding knowledge on what I have and also like he has said, our family many of them are drunkards, and I really wanted to help them. I have already started talking to some, and they have started dropping, not that they have dropped completely but at least. (FGD screeners, county 1)

Let’s say when we are here in the hospital, they believe in us according to the relationship we have outside the hospital. When in the hospital, we interact with many people and happen to socialize with them. That is an added advantage, especially to the screeners because we don‘t come from far, so those who meet with during the screening are our dear people. (FGD screeners, county 2)

Field observation also confirmed the motivation of screeners in private facilities. However, in private facilities, the screeners were not lay health workers from the communities where the patients were located and did not have as many opportunities to interact with and support participants after the initial screening.

#### Theme 4. Other Research Design Elements Potentially Supporting Change (***Table ***9)

##### Subtheme 4.A. Repeated Assessments by Research Staff

 Repeated assessments by research staff were seen by some RCT participants as educational opportunities and a potential source of support. The baseline and follow-up assessments included questions about alcohol use and related consequences such as sexual activities risks, theft, violence, housing, ability to work, depression, suicidality, quality of life, and self-stigma. Participants, especially in county 2, and some in private facilities, reported these questionnaires, and the support from the research staff helped them realize the impact of their substance use, decrease their use, and celebrate their progress, generating self-efficacy.

##### Subtheme 4.B. Soap Seen as an Incentive or Reward for Change and Staying Engaged

 RCT participants were given soap as a form of compensation for the time spent answering the research surveys administered by the research staff. Some RCT participants, especially in public facilities, attributed meanings to the provision of soap. They saw it as an encouragement to be “clean” and to take care of themselves and their families. Some mentioned perceiving the soap as an incentive to change, to stay engaged in the change process, or as a reward for decreasing their alcohol consumption. However, in the FGD Fb + BI private facilities, the soap was criticized, with participants wishing to receive something more valuable, like baseball caps or T-shirts, explaining they did not need soap. In public facilities, some participants would have been preferred food or money for transport to the healthcare facility.

## Discussion

In summary, this is one of the largest mixed-method studies of the effectiveness of blended-eLearning on health worker’s ability to support patients in decreasing harm from alcohol use, filling an important gap in the literature. Two RCTs (one in public and one in private facilities, total *n* = 1,205) and an extensive parallel qualitative study (field observations and 18 FGDs or KIIs with 117 participants) indicate that LMIC trainees (lay health workers, support staff or primary care clinicians) in public and private facilities, in outpatient departments and community clinics, in rural and urban areas, can successfully change their practice, decrease their stigma, and help participants decrease their alcohol consumption with significant improvements in patients’ lives. Other studies on blended-eLearning or eLearning rarely assess patient outcomes, and even less in such a comprehensive manner, and were mainly carried out on health sciences students, not practicing health workers, with only a few studies specific to LMICs (George et al., 2014; Hew & Cheung, 2014; Lewis et al., 2014; Liu et al., 2016; Maloney et al., 2012, 2015; Marrinan et al., 2015; Sandars, 2010; Shorbaji et al., 2015; Sinclair et al., 2015; Walsh et al., 2010).

A significant finding, supported by qualitative and quantitative results, is that motivated lay health workers and support staff, who completed a short blended-eLearning course, delivered an intervention with equivalent alcohol-related patient outcomes to clinician-led (physicians, nurses, or medical officers) additional BI. Other studies have demonstrated the effectiveness of lay health workers’ interventions to decrease alcohol use risk or improve other mental disorders (van Ginneken et al., 2021), but none by training health workers through free, blended-eLearning or eLearning, to our knowledge.

There is also a paucity of qualitative studies on lay health workers’ contributions to effective interventions (Glenton et al., 2011), which is another gap that is partially addressed by our study. The confounding factor from lay health workers going beyond the provision of the ASSIST results, by offering additional support in the community, limits the purity of assessment of the added BI’s effect by clinicians to the ASSIST screening and feedback. Support staff in private facilities could not provide that extra support in the community. Despite that, the alcohol consumption decrease was similar for those who received the Fb or the Fb + BI in private facilities, suggesting that extra community support is not a necessary component of effective intervention, above the delivery of the FRAMES components with decreased stigma.

The profound change in attitude and practice in the health workers and their entire clinic attracted new patients just for that service. This finding is key, as stigma and a lack of emphasis on the need to provide services to those with risky substance use contribute to the dismal access to services in both HIC and LMICs (Corrigan & Watson, 2002; Corrigan et al., 2004; Room, 2005; van Boekel et al., 2013). Factors promoting change from educational interventions also found influential in other studies, including personal motivation, support by the leadership, an opportunity to learn elements of BI, and being part of a group changing its attitude globally (Embleton et al., 2013; Morris & Chen, 2009; Sengwana & Puoane, 2004).

The decrease by more than 235 g/week at 6 months in all groups is of unprecedented magnitude (14 g being one alcohol serving). A Cochrane review found a mean intervention decrease of − 21.56 g/week (95% CI − 31.56, − 11.55) at 6 months and 20 g/week (95% CI − 28.36, − 11.8) at 1 year (Kaner et al., 2018). A few studies have reported a reduction of around 100 g (Kaner et al., 2018). Naturally, studies with higher baseline consumption may observe a larger decrease (Kaner et al., 2018). In our RCTs, the baseline consumption was high, averaging 326 to 423 g/week. That level was comparable to other studies with only men, who constitute more than 90% of our sample (Kaner et al., 2018).

Social desirability bias, which could have increased by the end of the study from all the attention paid to substance use issues and regression toward the mean, could have inflated the decrease in consumption (Dawson-Saunders & Trapp, 1994). Furthermore, some may find the measurement of reduced alcohol consumption in grams as an insufficient outcome to demonstrate a meaningful reduction in alcohol-related adverse consequences, believing that only sobriety matters as an outcome. However, findings from the field observation, FGDs and KIIs, with patients and health worker, in all settings and groups support the plausibility of a sizeable quantitative decrease in alcohol use resulting in a meaningful impact on patients’ lives.

Vast differences exist between related to patients and service providers in LMICs vs. HICs in terms of availability and level of training, access to primary care, general population level of awareness of substance use impacts, cultural norms, and social contexts (Awenva et al., 2010; Babor et al., 2013; O'Donnell et al., 2013). Such differences might have contributed to the unexpectedly much-larger decrease, considering the novelty of the intervention, newer expression of empathy, and a significant reduction in stigma, as well as tremendous new knowledge and skills transfer to patients, as observed in our qualitative findings.

Previous research showed that empathy is a strong mediator of the therapeutic effect (Moyers & Miller, 2013). The courses explicitly covered stigma and empathy, both in the written material, exercises, in-person demonstrations, and experiential learning activities, with some meant to trigger reflection on stigma, as per best practices to decrease stigma (Clair et al., 2019, in press; Livingston et al., 2012).

Furthermore, the first three FRAMES components (feedback, responsibility, and advice) are key to effective BI (Humeniuk et al., 2010a), and there was ample evidence that these three components were delivered to participants in all groups and settings. The design of the ASSIST screening, with its insightful questions, and the feedback form, which covers some elements of BI, seems to contribute to the delivery of those three components of FRAMES, per our qualitative findings. Other studies using the ASSIST screening tool have observed significant and similar decreases in both groups (Assanangkornchai et al., 2015), which supports that the ASSIST feedback tool can be effective. The repeated use of the ASSIST and other research surveys, at 3 and 6 months, might have generated further decrease after the initial intervention. Therefore, our studies provide some insight into improving methods for further trials. Further research could confirm if the delivery of the ASSIST results via its feedback form is an effective intervention. Also, since the soap seemed to motivate some participants, as a reward or as an encouraging gesture, it could be interesting to study such incentives as part of contingency management interventions for substance use in LMIC populations having difficulties accessing even such a basic necessity of life.

Another limitation is the small number of screeners in the private facilities with the FGD, including both screeners and clinicians and the lack of identifiers of who was speaking in the audio recording. However, field observations align with the interpretation of the FGDs and KIIs.

This study has several strengths, including that it was conducted with health workers in a variety of clinical settings where Kenyans usually seek care, addressing an important gap in studying intervention for alcohol use in LMICs. It is a large mixed-method study with rich inferential qualitative findings aiding the interpretation of the RCTs’ results, offering an opportunity for triangulating findings and contextualizing conditions that supported those results (Fortin et al., 2021; Pope et al., 2007; Spillman, 2014; Todd, et al., 2004). The loss to follow-up was small in public (7%) and larger (30%) in private facilities. Using intention-to-treat analysis with sensitivity analysis with or without imputation showed similar outcomes, congruent with a lack of a significant attrition bias. The 6-month follow-up period is also adequate, as per the Cochrane review, with showed minimal changes from 6 to 12 months (Kaner et al., 2018). Other strengths include that the randomization was carried out with high fidelity, with few participants in the Fb group receiving a formal BI by a clinician and appropriate concealment of group allocation during quantitative analysis. Furthermore, our study included qualitative researchers of multiple backgrounds, and the interpretation of the findings was corroborated with participating health workers and the research staff who carried out the field observations. In conclusion, and consistent with other studies (van Ginneken et al., 2021), our findings found that motivated lay health workers and support staff can learn and deliver BI as effectively as primary care clinicians when using tools such as the ASSIST report card and covering most FRAMES components. Furthermore, significant changes in health workers’ knowledge, skills, and stigmatizing vs. empathic attitudes led to significant reductions in alcohol overuse and meaningful patient life improvements. The acquisition of such transformative competencies through blended-eLearning courses was feasible even in an LMIC, regardless of facilities being private or public, outpatient department or community clinics, and in rural or urban areas. Educators can use the eDATA-K blended-eLearning NextGenU.org competency-based courses created through assembling freely available high quality online learning content with mentored and peer activities to teach effective screening and brief intervention. These findings suggest a major scale-up of human resources to address the significant burdens from risky alcohol use is feasible through blended-eLearning at multiple provider levels, including lay health workers, using the eDATA-K courses. In addition, the feasibility has been highlighted in the recent COVID-19 pandemic during which several universities have used these courses as they were forced to switch to online learning, which have been used by several universities when they needed to switch to online learning during the COVID-19 pandemic. The model could also potentially address other health priorities by creating other continuing education courses or health education curricula in collaboration with NextGenU.org.

## Supplementary Information

Below is the link to the electronic supplementary material.

Supplementary file1 (DOCX 37 kb)

Supplementary file2 (DOCX 24 kb)
