# Supplementary material for: Blended-eLearning Improves Alcohol Use Care in Kenya: Pragmatic Randomized Control Trial Results and Parallel Qualitative Study Implications
Source: Int J Ment Health Addict. 2022 Aug 12;20(6):3410–37. doi: 10.1007/s11469-022-00841-x (PMC9373889; doi:10.1007/s11469-022-00841-x)
Supplement: Supplementary file 1 — Supplementary file1 (DOCX 37 kb) [file 11469_2022_841_MOESM1_ESM.docx]

ASSIST alcohol related score ≥ 11 (moderate to high risk) offered to take part in a study on health risk

Meets inclusion/exclusion criteria: offered to participate

All patient ≥ 18 y.o. presenting to a participating facility are offered to partake in health screening by a screener

Screener administers consent for the screening study

Interested in participating: referred to study staff

ASSIST alcohol related score <11

patient is excluded

Not consenting: excluded; All excluded patient continue to wait for regular clinical consultation

Clinician to perform BI

and offer up to 2 follow-up consultations

Allocated to Fb group: asked to wait for regular consultation

Not consenting: excluded

Not meeting inclusion/exclusion criteria: excluded

Allocated to Fb+BI group: waits for consultation and asked to show and discuss the ASSIST feedback form to the clinician

Consents: randomized to Fb or Fb+BI group

and initial questionnaires administered

Not interested in participating: excluded

Follow-up research questionnaires administered at 1, 3 and 6 months by research staff not involved in randomization

Consenting: offered the screening with the ASSIST, RAPA, and BMI and provided brief feedback

Fb: Feedback group, Fb+BI: Feedback and brief intervention group

**Figure S.1: Randomized control trials procedure and flow**
