# Supplementary material for: Blended-eLearning Improves Alcohol Use Care in Kenya: Pragmatic Randomized Control Trial Results and Parallel Qualitative Study Implications
Source: Int J Ment Health Addict. 2022 Aug 12;20(6):3410–37. doi: 10.1007/s11469-022-00841-x (PMC9373889; doi:10.1007/s11469-022-00841-x)
Supplement: Supplementary file 2 — Supplementary file2 (DOCX 24 kb) [file 11469_2022_841_MOESM2_ESM.docx]

**Supplementary data**

**Table S.1. Socioeconomic status and changes in alcohol consumption in public and private facilities (Generalized Estimating Equations raw scores)**

|  |  | **Public** | | | | | **Private** | | | | |
| --- | --- | --- | --- | --- | --- | --- | --- | --- | --- | --- | --- |
| **Variable** | **Category** | **β** | **S.E.** | **95% Confidence**  **Interval** | | ***p*** | **β** | **S.E.** | **95% Confidence**  **Interval** | | ***p*** |
|  |  |  |  | **Lower** | **Upper** |  |  |  | **Lower** | **Upper** |  |
| Treatment allocation | Fb+BI | 13.40 | 48.60 | -81.86 | 108.65 | 0.783 | 70.91 | 45.3 | -17.92 | 159.74 | 0.118 |
|  | Fb | Ref. |  |  |  |  | Ref. |  |  |  |  |
| Gender | male | 52.80 | 20.49 | 12.64 | 92.96 | **0.010^a^** | 140.31 | 29.5 | 82.46 | 198.15 | **<0.001^a^** |
|  | female | Ref. |  |  |  |  | Ref. |  |  |  |  |
| Wealth index quintile | 1 | -69.44 | 23.11 | -114.73 | -24.15 | **0.003^a^** | 58.01 | 27.2 | 4.74 | 111.28 | **0.033^a^** |
|  | 2 | -38.20 | 24.37 | -85.97 | 9.56 | 0.117 | 65.68 | 22.1 | 22.35 | 109.02 | **0.003^a^** |
|  | 3 | 22.71 | 28.64 | -33.42 | 78.85 | 0.428 | 109.12 | 25.1 | 59.88 | 158.36 | **<0.001^a^** |
|  | 4 | 90.48 | 96.40 | -98.45 | 279.42 | 0.348 | 30.35 | 18.7 | -6.23 | 66.92 | 0.104 |
|  | 5 | Ref. |  |  |  |  | Ref. |  |  |  |  |
| Employment status | self-employed | 7.24 | 26.00 | -43.72 | 58.21 | 0.781 | -47.76 | 61.1 | -167.49 | 71.97 | 0.434 |
|  | casual labor | 44.23 | 26.16 | -7.04 | 95.49 | 0.091 | -29.90 | 67.0 | -161.20 | 101.40 | 0.655 |
|  | employed | 24.10 | 27.59 | -29.98 | 78.18 | 0.382 | -97.17 | 59.3 | -213.44 | 19.09 | 0.101 |
|  | unemployed | Ref. |  |  |  |  | Ref. |  |  |  |  |
| Marital status | ever married | 30.75 | 23.29 | -14.90 | 76.41 | 0.187 | 76.19 | 27.5 | 22.25 | 130.13 | **0.006^a^** |
|  | single | Ref. |  |  |  |  | Ref. |  |  |  |  |
| Education level | primary or less | -5.05 | 34.05 | -71.78 | 61.68 | 0.882 | 26.94 | 32.2 | -36.11 | 89.99 | 0.402 |
|  | secondary | -21.83 | 33.68 | -87.85 | 44.19 | 0.517 | -13.21 | 20.5 | -53.49 | 27.06 | 0.520 |
|  | tertiary | Ref. |  |  |  |  | Ref. |  |  |  |  |
| Household size | | -4.92 | 2.15 | -9.13 | -0.72 | **0.022^a^** | -0.56 | 3.9 | -8.13 | 7.00 | 0.884 |
| Age (years) | | -0.31 | 0.75 | -1.77 | 1.15 | 0.679 | -3.68 | 1.2 | -5.99 | -1.38 | **0.002^a^** |
| Time | | -102.68 | 10.13 | -122.55 | -82.82 | **<0.001^a^** | -84.78 | 9.2 | -102.80 | -66.76 | **<0.001^a^** |
| FB+BI *Time | | -9.09 | 15.30 | -39.07 | 20.90 | 0.553 | -18.01 | 13.4 | -44.21 | 8.19 | 0.178 |
| Feedback*Time | | Ref. |  |  |  |  | Ref. |  |  |  |  |

a: statistically significant at p<0.05
